# Km-scale coupled simulation and model–observation SST trend discrepancy

**DOI:** 10.1073/pnas.2522161123

**Published:** 2026-02-19

**Authors:** Sarah M. Kang, Dian A. Putrasahan, Noel G. Brizuela, Helmuth Haak, Jürgen Kröger, Jochem Marotzke, Bjorn Stevens, Jin-Song von Storch

**Affiliations:** ^a^Max Planck Institute for Meteorology, Hamburg, Germany

**Keywords:** km-scale climate models, tropical Pacific warming pattern, Southern Ocean cooling, ocean eddy heat transport, stratocumulus cloud feedback

## Abstract

The spatial pattern of sea surface temperature (SST) change in the tropical Pacific plays a central role in shaping global climate through its influence on atmospheric circulation, rainfall, and extreme weather. Accurately simulating this pattern is critical for reliable near-term climate projections, yet persistent discrepancies between models and observations remain. While global warming is unequivocal, satellite-era observations reveal pronounced cooling in the southeastern tropical Pacific and Southern Ocean, contrary to the warming simulated by most CMIP models. These discrepancies raise concerns about the reliability of current models in projecting near-term regional climate change. Here, we present the historical simulation with the ICON coupled model at a 5 km ocean and 10 km atmosphere grid-spacing. ICON successfully reproduces the observed SST trends, including cooling in both the Southern Ocean and the southeastern tropical Pacific. Its fidelity is enabled by the direct representation of eddy heat transport across the Antarctic Circumpolar fronts and realistic stratocumulus cloud feedbacks in the subtropical southeast Pacific. These findings highlight the importance of fine spatial resolution in capturing the mechanisms of heat uptake in the eddy-rich Southern Ocean. Our results suggest a pathway for resolving long-standing biases in historical simulations and improving confidence in near-term climate projections.

## Significance

Accurately simulating historical sea surface temperature (SST) trends is critical for reliable near-term climate projections. Yet, most climate models fail to capture the observed cooling in the Southern Ocean and southeastern tropical Pacific—two persistent model–observation discrepancies. Using a km-scale coupled simulation, we successfully reproduce these key features by explicitly resolving ocean eddies and simulating realistically strong stratocumulus cloud feedbacks. We argue that km-scale spatial resolution could improve representations of ocean heat uptake and extratropics-to-tropics teleconnections, potentially reducing long-standing model–observation discrepancies and increasing confidence in near-term climate projections.

During the satellite era, despite the global warming trend driven by increasing greenhouse gas concentrations, the tropical Pacific has experienced a pronounced decline in sea surface temperatures (SST), particularly in the southeastern basin (1). This observed pattern is characterized by a strengthened zonal SST gradient along the equator. In stark contrast, conventional Coupled Model Intercomparison Project (CMIP)-class models simulate basin-wide warming in the tropical Pacific accompanied by a weakening of the zonal SST gradient—contrary to observations (2). These opposing trends raise concerns that current models may misrepresent near-term projections of high-impact storm activity (3, 4) and regional terrestrial precipitation (5). The persistent model–observation discrepancy in tropical Pacific warming patterns further implies systematic biases in simulated climate feedbacks and climate sensitivity (6). Given that the tropical Pacific warming pattern exerts widespread influence through planetary-scale atmospheric wave responses (7), it plays a key role in shaping regional climate variability. Elucidating the physical mechanisms underlying the observed eastern Pacific cooling is therefore essential for improving the reliability of near-term climate projections and informing effective societal adaptation strategies.

The observed cooling trend in tropical Pacific SST has often been attributed to internal ocean–atmosphere variability (8). However, its persistence over recent decades, combined with the inability of multiple large-ensemble historical simulations to reproduce it (1), has led to growing recognition that external forcing may be at least partially responsible (9). Proposed drivers include geographically heterogeneous aerosol forcing, characterized by cooling over Eurasia and warming over North America and Europe (10, 11), stratospheric ozone depletion over Antarctica (12), interbasin interactions with Atlantic and/or Indian Ocean warming (13–15), and the ocean dynamical thermostat mechanism, whereby upwelling of cold waters leads to surface cooling (16–18).

Another salient feature absent in most models is the cooling of the Southern Ocean (1), which has been linked to the observed cooling in the southeastern tropical Pacific (19, 20). This high-latitude cooling may arise from a combination of Antarctic ozone depletion (21), natural multidecadal variability involving Southern Ocean convection (22), and freshwater input from ice-sheet melt (23). However, such multidecadal variability is often poorly represented, and Antarctic meltwater effects are missing in conventional climate models, likely explaining the absence of Southern Ocean cooling in historical simulations. Even when Southern Ocean cooling is prescribed, its teleconnection to the tropics remains muted in many models (24) due to weak stratocumulus cloud feedback in the subtropical southeast Pacific, which is critical for amplifying the signal (20).

A leading hypothesis for the anomalous Southern Ocean response in models is their inadequate representation of mesoscale ocean eddies. For example, the Southern Ocean circulation response to CO_2_-strengthened westerlies is determined by the competing effects of increased Ekman northward transport and counteracting mesoscale eddy fluxes (25). Previous simulations have found that ocean cooling driven by enhanced northward advection of polar waters can be largely offset and often surpassed by eddy-driven transport of warm subtropical water masses across the Antarctic Circumpolar Current (ACC) (26). A correct representation of these eddies is therefore essential for accurately simulating changes in ACC transport, Southern Ocean overturning, and stratification under CO_2_ forcing. Explicitly representing mesoscale eddies in the Southern Ocean requires a minimum spatial resolution of approximately 10 km (27). Indeed, coupled models with 0.1° ocean and 0.5° atmosphere resolution, which explicitly resolve these features, have shown improved representation of Southern Ocean SST variability (28, 29). Nonetheless, higher resolution alone does not ensure better performance in simulating historical Southern Ocean cooling trends. An analysis of seven models participating in High-Resolution Model Intercomparison Project (HighResMIP) (30) indicates that increasing horizontal resolution does not consistently resolve the discrepancy in historical warming patterns (31). This may reflect the fact that, in some HighResMIP models, particularly those with ocean grids of 0.25°, the resolution remains insufficient to properly represent mesoscale eddy dynamics in the Southern Ocean.

**Fig. 1.**
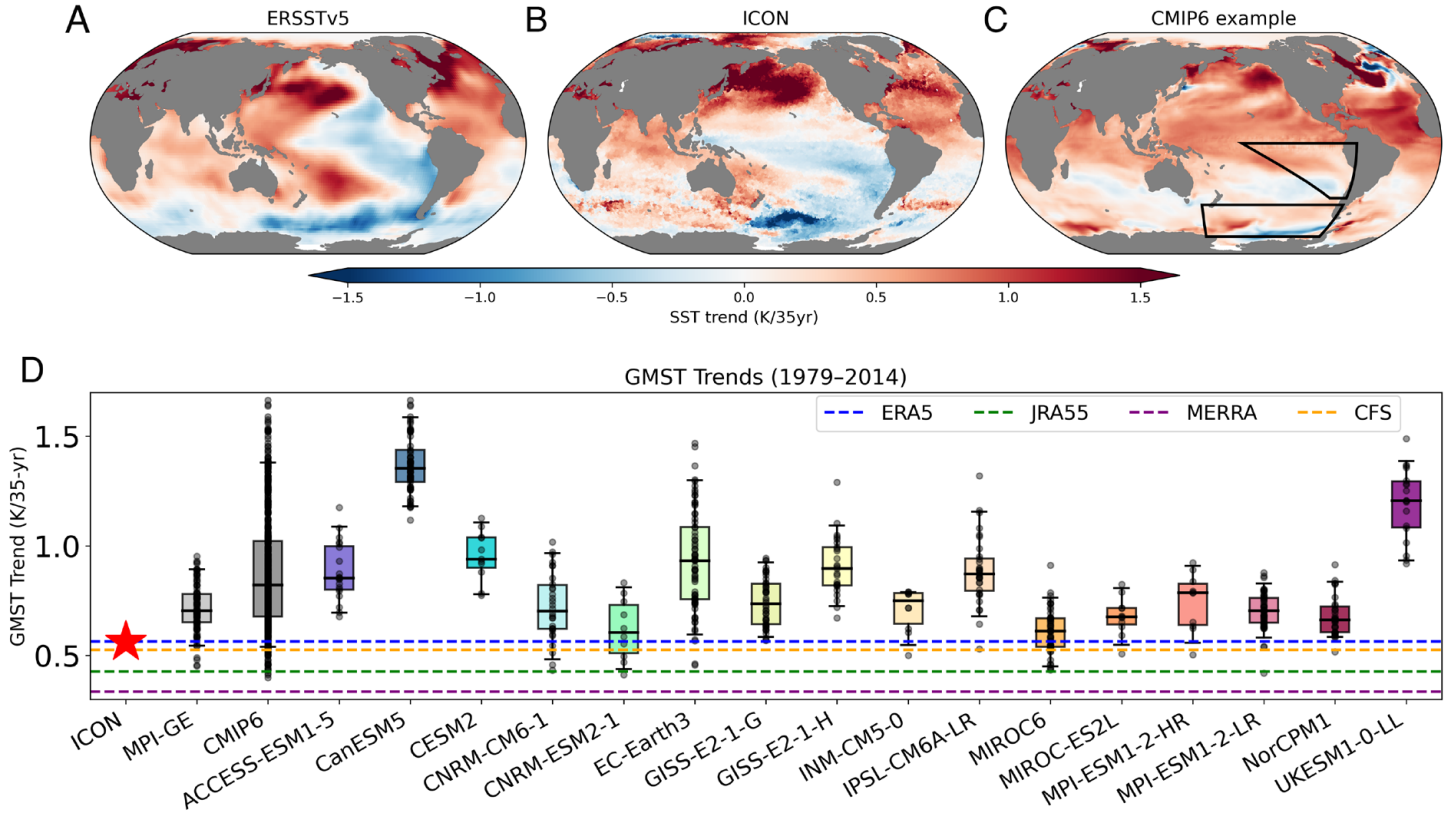
Comparison of surface temperature trends over 1979 to 2014 in observations, ICON, and CMIP6 ensemble. Spatial maps of SST trend based on (*A*) ERSSTv5 observations (33), (*B*) ICON historical simulation, (*C*) one of the CMIP6 realizations close to CMIP6 multimodel mean (black circle in Fig. 2). (*D*) Box-whisker plot of global mean surface temperature (GMST) trends for 100-member MPI-ESM Grand Ensemble (MPI-GE), all available CMIP6, and subgroups of CMIP6 ensembles that have 10 members or more of their particular model. Solid black line within each box mark 50% percentile, edges of the box represent 25% and 75% percentile, and whiskers mark 5% and 95% percentile. Gray circles represent individual ensemble members. The GMST trend from ICON (indicated by the red star and red solid line) can be compared with multiple observational products, as shown in the legend.

Here, we demonstrate that a historical simulation using the Sapphire configuration of the ICON model (*Materials and Methods*), with resolution of 5 km in the ocean and 10 km in the atmosphere (32), reproduces the observed SST trends with remarkable fidelity, capturing both the pronounced cooling in the southeastern tropical Pacific and the Southern Ocean (Fig. 1 *A* and *B* and *SI Appendix*, Fig. S1). Unlike CMIP-class models, this fine-scale resolution enables the explicit representation of deep atmospheric convection and mesoscale ocean eddies. Directly resolved vertical eddy heat transport along the ACC fronts enables dynamical adjustments that enhance ocean heat uptake and export, ultimately cooling the Pacific sector of the Southern Ocean. This cooling, in turn, triggers a teleconnection to the tropical Pacific, mediated by sufficiently strong positive cloud feedback in the subtropical southeast Pacific. This represents the coupled historical simulation at such high resolution that successfully reproduces these key observed features, offering insight into the dynamics linking high-latitude and tropical climate change.

## Performance of ICON Historical Simulation.

**Fig. 2.**
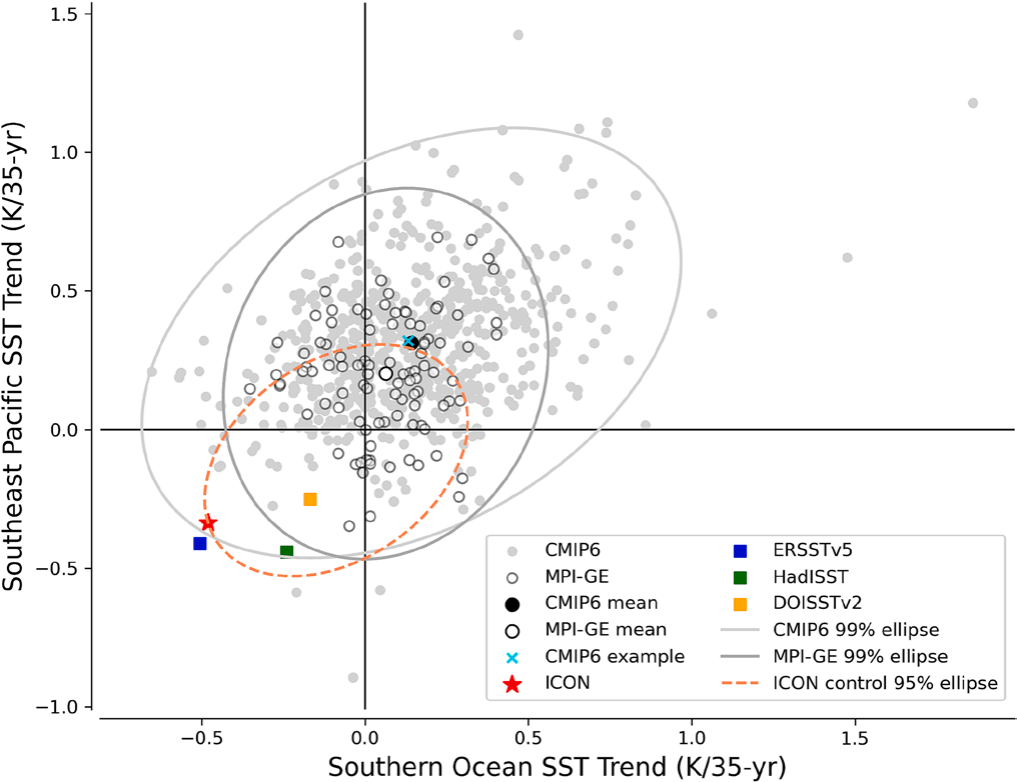
Comparison between Southern Ocean SST trends and Southeast Pacific SST trends. Scatter plot of SST trends over Southern Ocean Pacific sector (black box in Fig. 1*C*) against Southeast Pacific (black triangle in Fig. 1*C*) for multiple observational datasets, ICON, MPI-GE, and CMIP6. A representative CMIP ensemble member used in Fig. 1*C* is marked with a cyan cross. The 99% confidence ellipse based on the Mahalanobis distance is shown in light gray for CMIP data and in dark gray for MPI-GE. The 95% confidence ellipse in the ICON control simulation is shown as a pink dashed line, constructed from 5,000 samples of trends computed from randomly selected 36-year segments from the 110-year ICON control simulation.

Spatial maps of SST trends reveal a striking resemblance between ICON and observations, in sharp contrast to CMIP-class models (Fig. 1). We focus on the period over 1979 to 2014, which is fully covered by the CMIP6 historical simulations (34). ICON, based on a single ensemble member, captures the pronounced cooling over the Southern Ocean and the southeastern tropical Pacific, extending into the equatorial region, closely mirroring the observed pattern. In contrast, a representative CMIP ensemble member, selected to align closely with the multimodel mean SST trends in the southeastern Pacific and Southern Ocean (two key regions marked in Fig. 1*C* and highlighted as a cyan cross in Fig. 2), exhibits widespread warming across the tropical Pacific and little to no cooling in the Southern Hemisphere high latitudes. This reflects a well-documented bias in CMIP-class models. While the CMIP multimodel mean (*SI Appendix*, Fig. S2) is commonly compared with observations, we present a representative ensemble member here, as it provides a more physically consistent and comparable basis for evaluating model performance against observations. The close agreement between the single-member ICON simulation and observations is particularly notable given the low likelihood of CMIP models reproducing the observed magnitude of regional SST trends (Fig. 2). Because the CMIP multimodel data combine both model structural uncertainty and internal variability, we also show the spread from the MPI-ESM Grand Ensemble (MPI-GE; Figs. 1*D* and 2) to isolate the range arising from internal variability alone. Although the MPI-ESM is among the CMIP models that best captures the observed patterns, such close agreement remains rare even across large ensembles. In particular, the ICON signal lies outside the 99% ellipse of the MPI-GE and only barely within the 99% ellipse of the CMIP models, as determined using the Mahalanobis distance—a multivariate metric that measures how far a point deviates from the joint distribution of reference data while accounting for correlations among variables (dark and light gray ellipses in Fig. 2). This indicates that, despite being a single realization, the ICON simulation likely behaves distinctly from conventional models.

To further assess the probability of ICON simulating a 35-year trend reaching the observed cooling amplitude over the two key regions, we use a 110-year control integration under fixed 1950 conditions following a 40-year spin-up. Due to the limited length of the control simulation, we construct synthetic 36-year segments by randomly sampling from the control run. A pink ellipse in Fig. 2, representing the 95% confidence region around the mean based on the Mahalanobis distance, is skewed toward the third quadrant, indicating that strong cooling trends occur slightly more frequently than strong warming trends in both regions under natural variability. However, the amplitude of cooling in the historical simulation (red star) is extremely rare within the control distribution. Based on the Mahalanobis distance, the ICON trends lie barely within the 95% ellipse of the ICON control distribution, suggesting that internal variability alone is unlikely to explain the simulated historical trend.

Extending the observational record to 2024 provides further insight into the persistence of regional SST trends. While observed tropical Pacific cooling is largely symmetric about the equator during 1979 to 2014 (Fig. 1*A*), the post-2014 trends show that cooling persists in the southeastern Pacific and the Southern Ocean, but weakens north of the equator (*SI Appendix*, Fig. S1*A*). This suggests that the North Pacific signal is more likely driven by internal variability, possibly associated with the Pacific Decadal Oscillation, rather than by external forcing. In contrast, the more persistent Southern Hemisphere cooling points to the possibility of a forced component rather than being purely internally driven. This observed shift in the northeastern Pacific SST trend makes the ICON historical simulation better aligned with observations over the full 1979 to 2024 period compared to 1979 to 2014 (contrast Fig. 1 *A* and *B* with *SI Appendix*, Fig. S1).

The inability of CMIP-class models to reproduce the observed cooling trends in the Southern Ocean and the tropical Pacific leads to an overestimation of historical global warming (Fig. 1*D*). In contrast, global land surface temperature trends are generally well captured (*SI Appendix*, Fig. S3). The ICON simulation yields a global-mean surface temperature increase of 0.56 K over the 35-year period, closely matching the amplitude observed in ERA5 (35). Given the substantial influence on global-mean surface temperature trends, it is necessary to resolve the persistent biases in the southeastern tropical Pacific and the Southern Ocean.

## Mechanism for Southern Ocean Cooling.

**Fig. 3.**
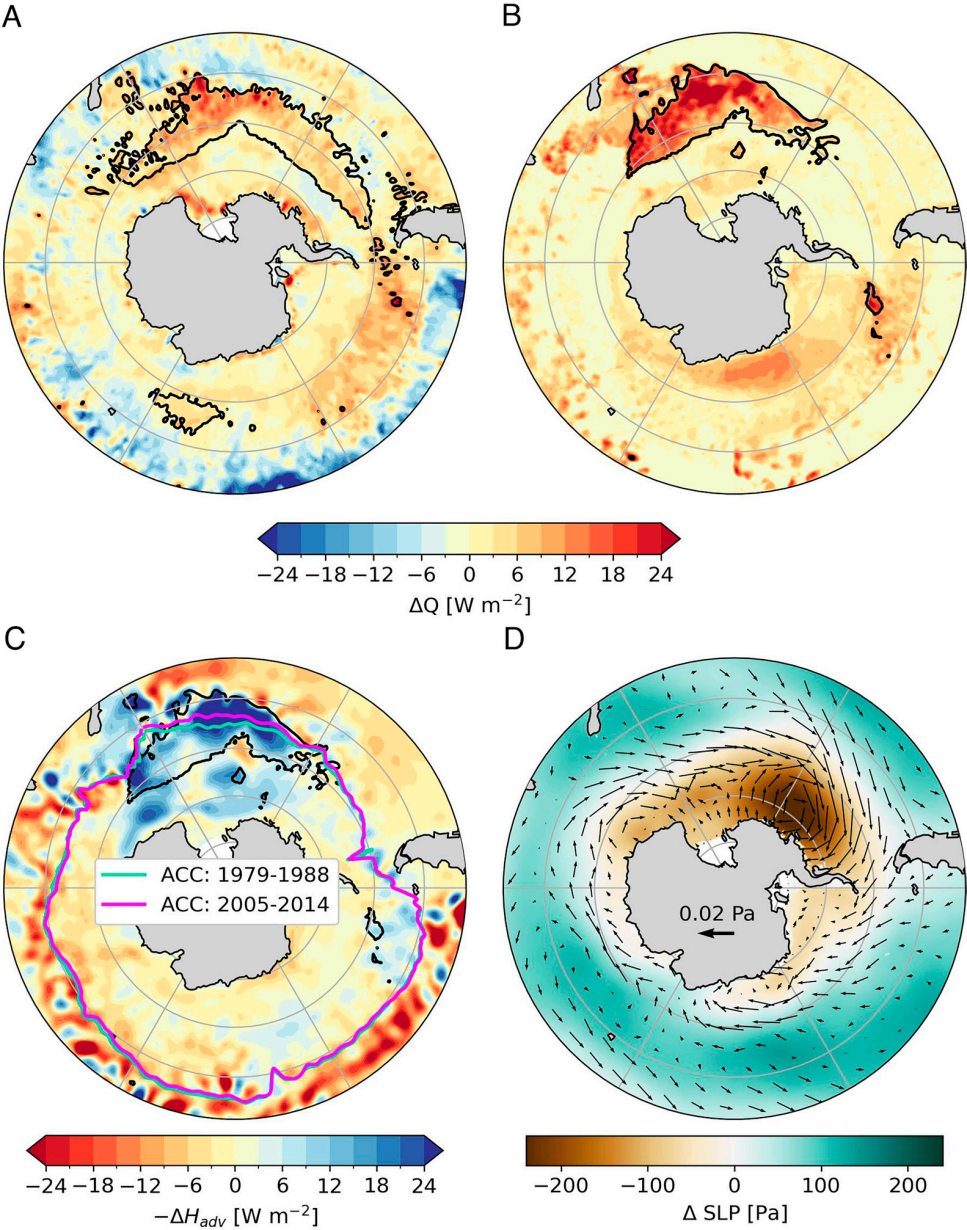
Changes between the two decades 2005 to 2014 and 1979 to 1988 in net downward surface heat flux in (*A*) ERA5 and (*B*) ICON. (*C*) ICON vertically integrated rates of heating implied by ocean heat transport (shading, see *Materials and Methods*), along with the latitudinal position of the ACC front in the earlier (green) and later (purple) decades. (*D*) ICON sea level pressure (shading), along with the horizontal viscous stress at the ocean surface. Black contour in *A*–*C* indicates regions where the SST cooling between the two decades exceeds 0.5 K.

**Fig. 4.**
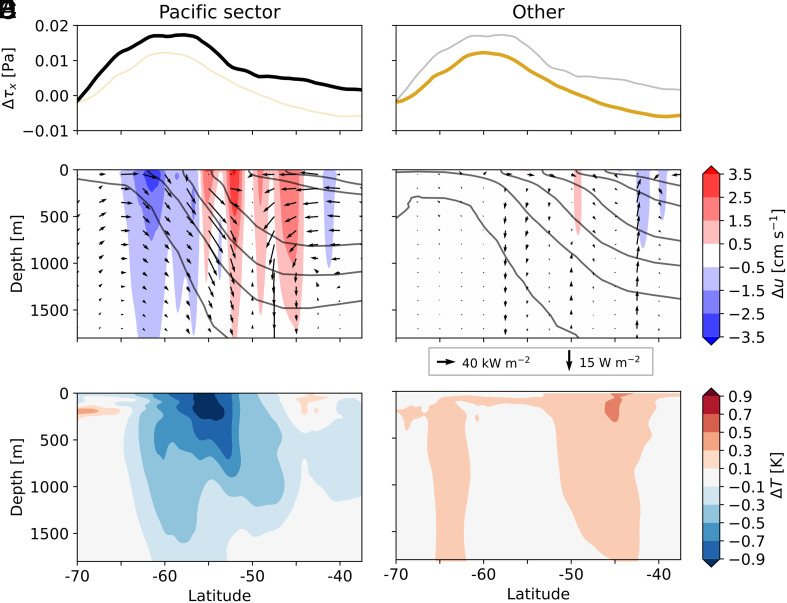
Zonally averaged ocean adjustments in the Southern Ocean between the decades 2005 to 2014 and 1979 to 1988. Changes in (*A* and *B*) zonal wind stress $\Delta \tau_{x}$, (*C* and *D*) zonal currents (shading) and meridional and vertical heat fluxes (vectors), and (*E* and *F*) subsurface ocean temperature (*Left*) within and (*Right*) outside the Pacific sector. Solid lines in (*C* and *D*) show the climatological location of isopycnals.

Based on ERA5, pronounced cooling of Southern Ocean SSTs occurs primarily in the Pacific sector, despite increased ocean heating from atmospheric fluxes ΔQ (Fig. 3*A*). A similar alignment between regions of positive ΔQ and substantial SST cooling is also found in ICON (Fig. 3*B*). This cooling extends throughout the water column (Fig. 4*E*), indicating that it must result from lateral heat export by ocean currents (Fig. 3*C*). Dynamical adjustments, rather than passive advection, are necessary to cool the region that receives increasing amount of heat. The clearest indicative feature of such adjustments is a local, northward displacement of the ACC (Fig. 3*C*), which reflects an expansion of the outcropping portion of polar water masses that are brought to the surface by wind-driven upwelling.

The zonal wind stress $\tau_{x}$ strengthens across the Southern Ocean, but this strengthening is up to 80% greater in the Pacific sector than elsewhere (Fig. 4 *A* and *B*), as the Amundsen Sea Low deepens by roughly 3 hPa (Fig. 3*D*). Similarly, changes in three-dimensional heat transport are markedly asymmetric, with substantial differences between the Pacific sector and other longitudes (arrows in Fig. 4 *C* and *D*).

Anomalous meridional heat fluxes toward regions of intensified ACC transport ensure that excess atmospheric heating is advected away from the Pacific sector and redistributed into other basins. Northward heat flux anomalies south of the ACC are partly driven by enhanced atmospheric heating and the Ekman response to strengthened zonal winds, while the downward heat fluxes following isopycnals in Fig. 4*C* indicate a reduction in the climatological poleward heat transport by mesoscale eddies (*SI Appendix*, Fig. S4) (36). Unable to cross the ACC fronts, the heat that is typically transported poleward by eddies is instead advected eastward by the ACC.

As the southern half of the ACC cools in response to reduced poleward heat transport by both eddies and the mean circulation (Fig. 4*C* and *SI Appendix*, Fig. S4), local sea surface height decreases, weakening the pressure gradients associated with the ACC and leading to a northward shift in its position (colored contours in Fig. 3*C*). With eastward currents strengthening north of 55°S (red shading in Fig. 4*C*), subtropical heat is advected away before eddy activity could help it penetrate poleward across ACC fronts. Ultimately, anomalous heat transport in the Pacific Sector is directed toward latitudes where the ACC intensifies, allowing excess heat supplied by ΔQ to be efficiently exported to other ocean basins. No such pattern is evident outside the Southern Ocean’s cooled region, where the ACC stays fixed or shifts poleward in line with net ocean warming (37) (Fig. 4 *D* and *F*). While passive advection by undisturbed Southern Ocean currents has been suggested as sufficient to delay warming in this basin (38), our results emphasize that dynamical adjustments are essential to explain the full-depth cooling in our simulation. Complete coupling between the eddy-rich ocean and atmosphere as well as zonally asymmetric strengthening of the surface wind stress (Figs. 3*D* and 4*A*) appear necessary to shift the ACC location and drastically increase heat export out of this one region of the Southern Ocean (Fig. 3*C*).

## Equatorward Teleconnection and Stratocumulus Cloud Feedback.

**Fig. 5.**
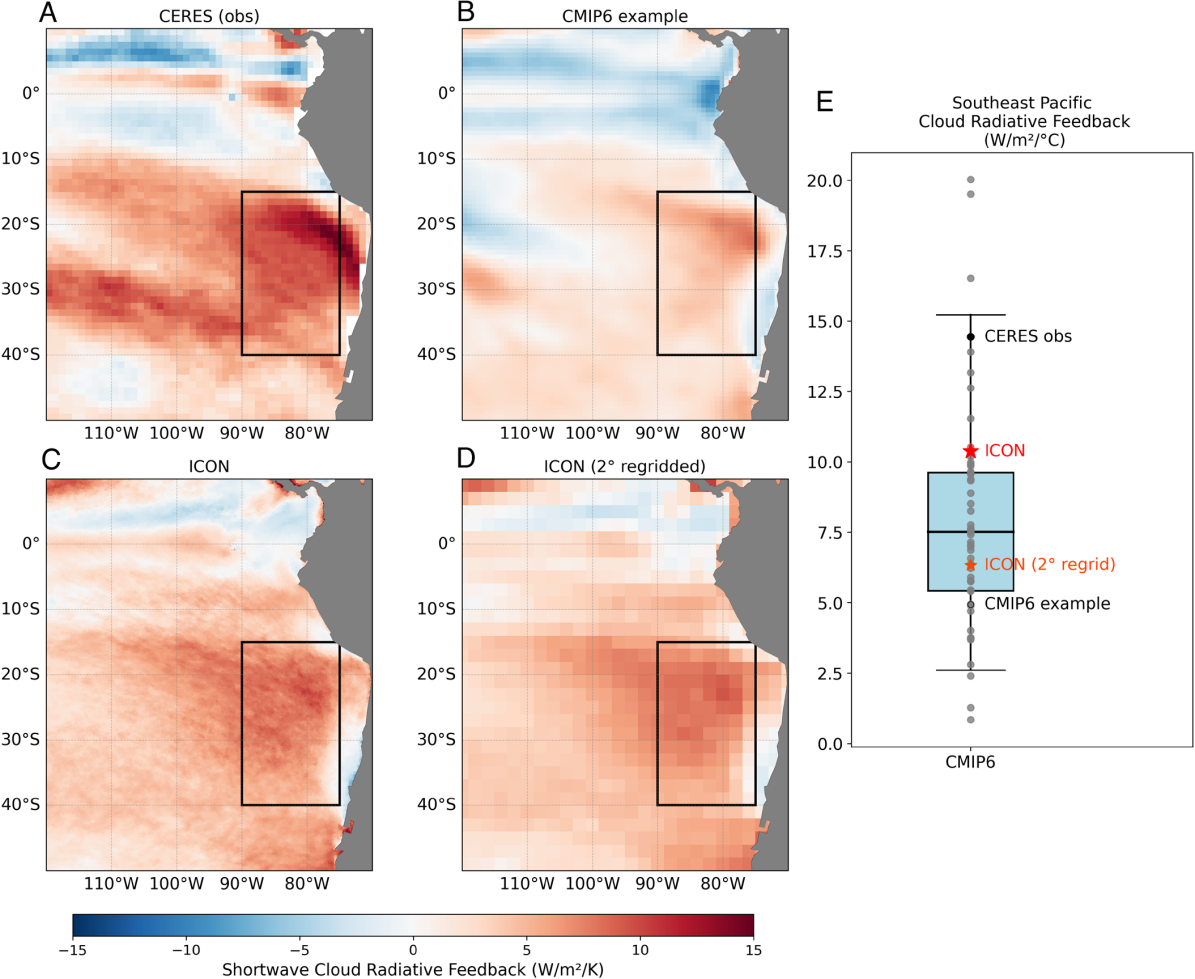
Coupling between SST and shortwave cloud radiative effect over the Southeast Pacific. Spatial distribution of shortwave cloud radiative effect regressed onto local SST (W m^−2^ K^−1^), with both variables detrended and deseasonalized, in (*A*) observations [radiative fluxes from the Clouds and Earth’s Radiant Energy Systems Energy Balanced and Filled data version 4.1 (42) and SST from ERSSTv5 (33)], (*B*) one of the CMIP6 models shown in Fig. 1*C*, (*C*) ICON model, and (*D*) panel *C* smoothed to 2° × 2° grid, and (*E*) the spatial average over the Southeast Pacific box (black rectangles in *A*–*D*).

Southern Ocean cooling occurs preferentially in the Pacific sector, where it exerts a strong influence on the tropical eastern Pacific (39). The cooling signal propagates equatorward following the climatological southeasterly flow. A high-pressure anomaly associated with the cooling strengthens these southeasterlies (Fig. 3*D* and *SI Appendix*, Fig. S5*A*), enhancing surface evaporation and thereby reinforcing the cooling through a wind–evaporation–SST feedback (40). As SSTs decline along the coasts of Chile and Peru, regions characterized by extensive stratocumulus cloud cover, lower-tropospheric stability increases, which in turn promotes enhanced cloud formation (20, 41) (Fig. 5 and *SI Appendix*, Fig. S5 *B* and *C*). The resulting increase in shortwave reflection further amplifies the cooling, establishing a coupled cloud–SST feedback. In most CMIP models, this feedback is too weak (Fig. 5*E*), limiting the extent to which Southern Ocean cooling influences the tropical Pacific. By contrast, in models with realistically strong cloud feedback, Southern Ocean cooling robustly induces cooling in the southeastern tropical Pacific (19, 20, 41). That is, the strength of the cloud feedback serves as a key mediator of the Southern Ocean–tropical Pacific teleconnection (20). In ICON, the cloud–SST coupling is 33% weaker than observed but lies on the stronger end of the CMIP model distribution (Fig. 5*E*). The sufficiently strong cloud feedback enables the equatorward propagation of the Southern Ocean cooling signal in ICON, ultimately contributing to the simulated southeastern Pacific cooling.

The relatively strong cloud feedback in ICON, compared to the majority of CMIP models, warrants further investigation. One possible explanation is that the fine spatial resolution allows localized cloud feedback to reach larger amplitudes at individual grid points, thereby sustaining a stronger regional average. This interpretation is supported by a sensitivity test in which the cloud feedback field is smoothed to a 2° × 2° grid, resulting in a 39% reduction in amplitude over the southeastern Pacific (Fig. 5 *D* and *E*). In addition, improved representation of terrain, such as the Andes Mountains, acts to block warm advection from the South American interior toward the coast, helping to maintain the low-level inversion structure that supports stratocumulus cloud formation (43, 44). The Peruvian stratocumulus region is particularly sensitive to SST variations, unlike other subtropical stratocumulus regions that are more influenced by free-tropospheric conditions (45), making accurate SST representation especially important. High-resolution topography (*SI Appendix*, Fig. S6) contributes to more realistic SSTs in the near-coastal eastern Pacific by improving the simulation of coastal wind systems and associated wind-driven upwelling (44). Notably, stronger cloud feedback in this region has also been reported in another high-resolution model with comparable resolution (0.25° atmosphere and 0.1° ocean) (44). Furthermore, models with even finer resolution at about 5 km in the atmosphere have been shown to realistically simulate the seasonal cycle of albedo and vertical structure of subtropical stratocumulus clouds (46).

## Discussion

**Fig. 6.**
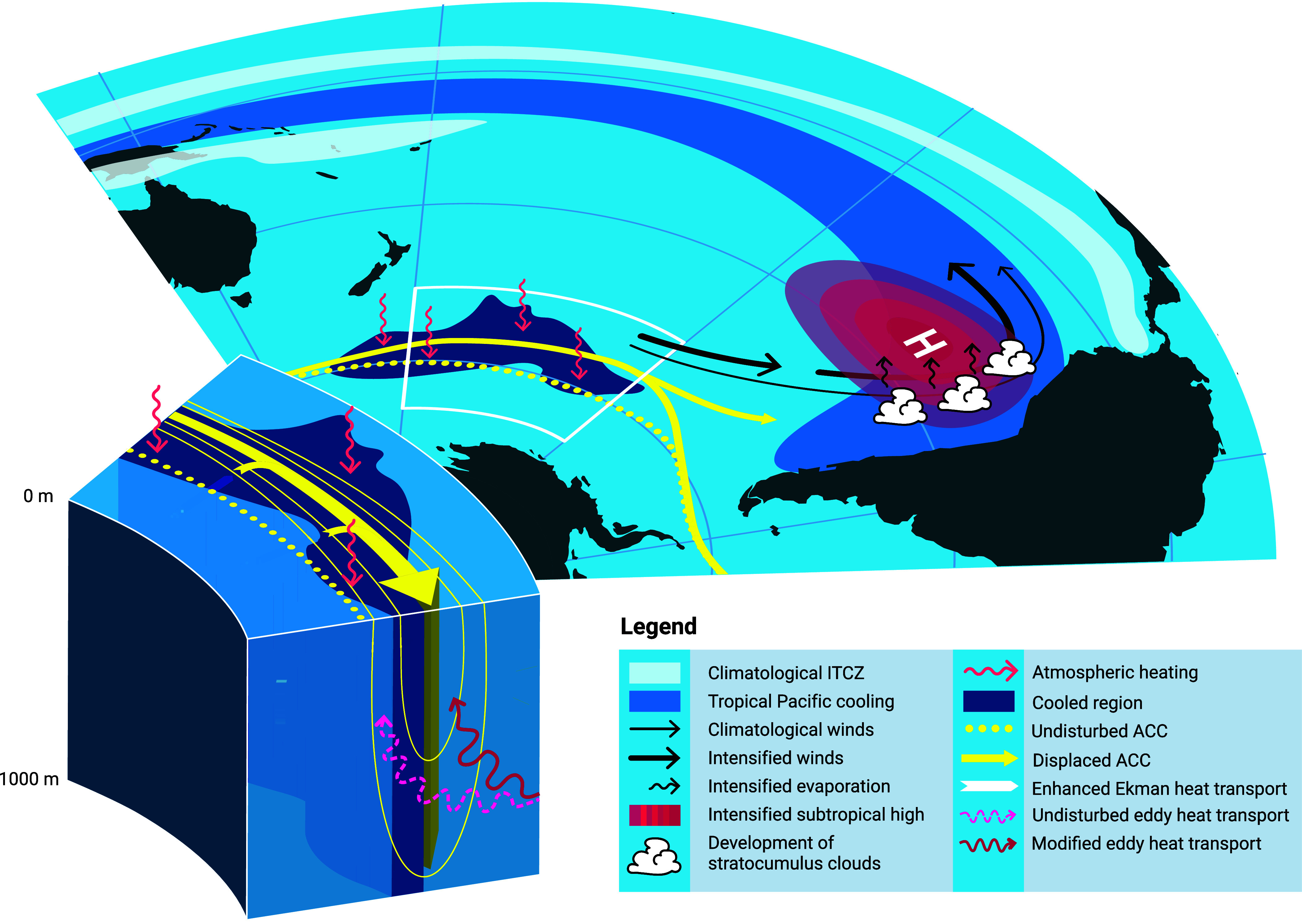
Schematic view of processes leading to improved SST trends in ICON. The lower-left inset illustrates dynamical ocean adjustments that enable polar water masses to shift equatorward while reducing poleward eddy heat fluxes across ACC fronts. Anomalous heat transport is ultimately directed toward a northward-shifted ACC, which exports excess heat away and into other basins. The mechanism by which Southern Ocean cooling is communicated to the tropical Pacific is described in ref. 20. Southern Ocean cooling is advected equatorward by the climatological southeasterlies. Anomalous high pressure associated with the cooling intensifies climatological wind speeds, enhancing evaporative cooling. Colder SSTs along the coast of Peru promote increased low cloud development, which in turn reflects more shortwave radiation, amplifying the cooling signal. As the cooling reaches the equatorial Pacific, it extends westward through the Bjerknes feedback, with the climatological ITCZ guiding its propagation.

We demonstrate that the km-scale ICON historical simulation captures the prominent Southern Ocean cooling, which in turn drives cooling in the southeastern tropical Pacific via sufficiently strong subtropical cloud feedback, closely resembling the observed pattern—one that CMIP-class models have notoriously failed to reproduce. The mechanism of Southern Ocean cooling and its teleconnection to the tropical Pacific is illustrated in Fig. 6. While we propose a plausible Southern Ocean-driven teleconnection, interactions between the Southern Ocean and the tropical Pacific are likely to be two-way coupled (39). The deepening of the Amundsen Sea Low—the key driver of Southern Ocean cooling in the Pacific sector—can originate from Rossby wave trains emanating from the tropics (47). However, the Amundsen Sea Low deepening can also arise independently from radiative forcing, particularly due to stratospheric ozone depletion (48, 49). It is also possible that the interaction between the changing atmospheric circulation and the high-resolution orography in our model contributed to a further deepening of the Amundsen Sea Low compared with conventional models. Without more targeted experiments, such as regional pacemaker simulations (19, 50), it remains challenging to definitively identify the trigger of the teleconnection. However, the southeastern tropical Pacific cooling is more likely initiated by the Southern Ocean than the reverse, as improvements in prediction skill in that region in a high-resolution model, relative to its low-resolution counterpart, have been attributed to enhanced prediction skills in the Southern Ocean (29). A growing body of literature points to the Southern Ocean as a key pacemaker of multidecadal variability in the tropical Pacific (19, 20, 29, 39, 51).

While it is very rare, some CMIP6 models do simulate cooling trends in our focus regions (Fig. 2 and *SI Appendix*, Fig. S2). However, if the Southern Ocean cooling is as strong as indicated by ERSSTv5, virtually no model captures both the Southern Ocean and Southeast Pacific cooling seen in the observations. Out of 714 members (i.e., 100 from MPI-GE and 614 from CMIP), fewer than ten capture the combined trend represented by the HADISST and DOISSTv2 datasets. Moreover, the ICON trends lie outside the range of variability in the control simulation (Fig. 2), suggesting that they may be more forced than internally driven, unlike the rare CMIP cases that likely occur by chance. Thus, despite being based on a single realization, the ICON simulation appears to behave distinctly from conventional models. Nevertheless, with only one ensemble member, it remains uncertain where this simulation would fall within the full ensemble spread, and we cannot yet confirm whether the ICON truly differs from the CMIP6 models. Even so, by creating such a realization, the ICON simulation allows us to identify a distinct underlying mechanism, as CMIP models do not explicitly resolve ocean mesoscale eddies—a central element of our proposed mechanism.

Our coupled historical simulation at 5 km ocean and 10 km atmosphere resolution potentially helps resolve a long-standing puzzle in historical SST trend patterns. However, km-scale models are not a universal solution. While many mean-state biases are reduced, others persist (32, 44). For example, the ICON model suffers from a significant cold bias along the equatorial Pacific (*SI Appendix*, Fig. S7) that leads to a pronounced dry bias in the western Pacific warm pool (*SI Appendix*, Fig. S8), which may explain why the southeastern Pacific cooling extends too far westward, reaching the Maritime Continent (Fig. 1*B*). The cold bias over the convective region is likely related to the prevalence of low clouds near the equator and the extent of ice clouds that warm the troposphere, both of which are sensitive to the model’s microphysical formulations. Nevertheless, two key advances have only become accessible through km-scale coupled modeling: the explicit representation of eddy heat transport across ACC fronts, essential for driving Southern Ocean cooling, and realistically strong stratocumulus cloud feedbacks, critical for propagating Southern Ocean-driven teleconnections to the tropics. We do not claim that km-scale simulations provide a definitive solution to the model–observation discrepancy. Km-scale models are themselves likely to exhibit model spread. Rather, our study aims to highlight that processes not represented in conventional models can play an important role in shaping the observed SST trends.

**Fig. 7.**
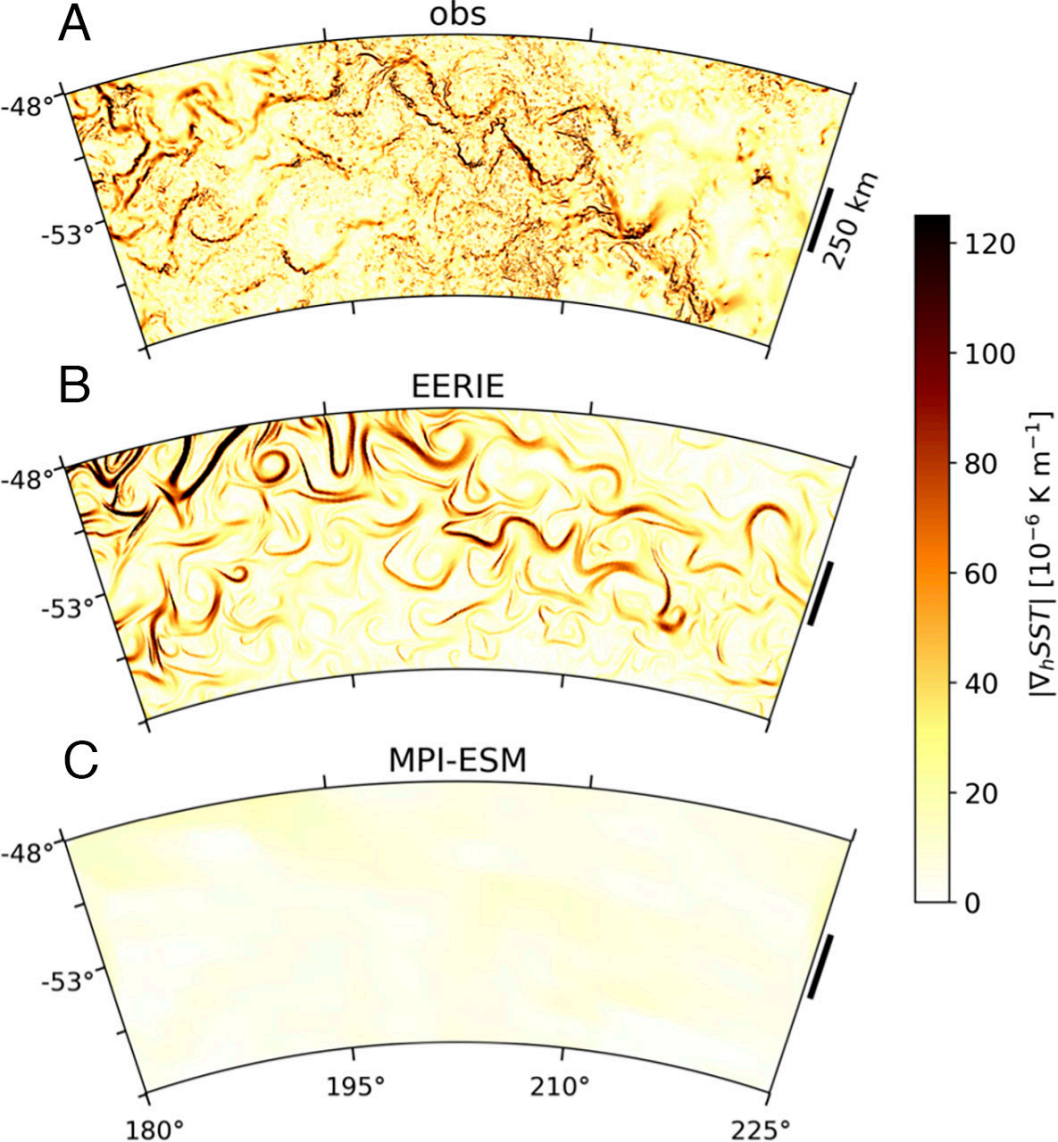
The signature of mesoscale ocean eddies. Snapshots of SST gradient amplitude on a random day in March from (*A*) the Multiscale Ultra High–Resolution SST product (53), (*B*) ICON, and (*C*) MPI-ESM participated in CMIP6. Observational data are from March 21 of 2011.

In particular, the direct and realistic representation of mesoscale ocean eddies (Fig. 7) allows heat uptake and transport in the Southern Ocean to respond dynamically to coupled ocean–atmosphere conditions. As described above, enhanced Ekman overturning, a northward shift in mesoscale eddy heat transport, and a northward-shifted ACC all contribute to exporting heat away from the Pacific sector of the Southern Ocean (Figs. 3*C* and 4). This ultimately improves the representation of the SST trend pattern (Figs. 1*B* and 2) and reduces the warm bias in global-mean surface temperature trends (Fig. 1*D*), given that the Southern Ocean accounts for ~70% of anthropogenic heat uptake (52). Subduction of anthropogenic heat by mesoscale eddies is a major driver of heat uptake in the Southern Ocean (36), and our ICON simulation resolves ACC thermal fronts where subduction occurs with a fidelity unattainable in conventional models (Fig. 7). Improved rates of Southern Ocean and global surface warming in our eddy-resolving ICON simulation thus point to the critical role of resolved mesoscale ocean processes in capturing the observed patterns of global climate change.

Our findings align with previous studies showing that explicitly simulating mesoscale eddies makes the Southern Ocean more effective at moderating anthropogenic warming around Antarctica, thereby delaying sea-ice decline (54). Delayed Southern Ocean warming has been proposed to contribute to the transition from the observed La Niña-like pattern, characterized by eastern Pacific cooling, toward a future El Niño-like warming pattern in the tropical Pacific (9, 55). Since km-scale models suggest an even more delayed Southern Ocean warming compared to standard CMIP-class models, the transition to an El Niño-like pattern may occur later than projected by conventional models.

## Materials and Methods

### ICON Simulations.

The ICON control and historical integrations are performed as part of the European project EERIE (https://eerie-project.eu), which aims at building a new generation of Earth System Models capable of explicitly representing an important, yet under explored regime of the Earth system—the ocean mesoscales also known as the ocean “weather”, using a model configuration developed by the European Project NextGEMS, which is based on the configuration described as Sapphire (32). The coupled version, which has a nominal grid spacing of 10 km in the atmosphere and 5 km in the ocean, is close to that used in ref. 32. Parameterizations of gray-zone processes, such as deep convection, orographic and nonorographic waves in the atmosphere, and mesoscale eddies in the ocean, which are partially but not completely resolved, are deactivated. The ICON simulations follow the HighResMIP protocol (30), meaning that both the control and the historical runs are branched off from a spin-up run under the 1950 conditions. In our case, the spin-up run lasts 40 y.

### Ocean Diagnostics.

Three-dimensional components of oceanic heat transport are computed online and saved as monthly averages of the products uT,vT, and wT. Rates of full-column heating implied by transport in Fig. 3*B* are computed as the full-depth vertical integral $H_{\mathrm{adv}}=\rho_{0}c_{p}\int \nabla \cdot \left(\bar{\mathrm{uT}},\bar{\mathrm{vT}}\right)dz$, where $\rho_{0}=1,025$ kg m^−3^, $c_{p}=3,900$ J K^−1^ kg^−1^, and the overline indicates a monthly average. Estimates of eddy temperature transport components (*SI Appendix*, Fig. S3) are computed for every month in our simulation as the difference $\overset{¯}{v^{\prime }T^{\prime }}=\overset{¯}{\mathrm{vT}}-\overset{¯}{v}\overset{¯}{T}$, such that they account for all submonthly covariance between simulated currents and temperature.

Lines denoting the mean ACC location in Fig. 3*B* are computed as the latitude of mean transport, following (37). Barotropic, geostrophic velocities $u_{g}$ are computed using the geostrophic relation and the model’s monthly sea surface height output. The mean latitude of transport is then computed as $\theta_{mean}=\int_{\theta_{S}}^{\theta_{N}}u_{g}\theta d\theta /\int_{\theta_{S}}^{\theta_{N}}u_{g}d\theta$, where integration limits $\theta_{S}$ and $\theta_{N}$ are computed separately for every month and longitude value. Integration limits are chosen to contain 90% of all eastward transport south of 35°S. Monthly values of $\theta_{mean}$ are then averaged for the decades shown in Fig. 3*B*. The northward shift in this location of mean ACC transport is found to be robust to the definition of integration limits and to occur in both winter and summer.

## Supplementary Material

Appendix 01 (PDF)

## Data Availability

Simulations were performed with the ICON branch feature-eerie-config as commit 8faad159. This source code is available here: 10.17617/3.IAZV21 (56). The ICON model is available to individuals under licenses adhering to BSD-3-Clause. By downloading the ICON source code, the user accepts the license agreement. Scripts employed to produce the figures are available here: 10.17617/3.0LKCBV (57), under the license terms of CC BY 4.0. Open access funding provided by the Max Planck Society.
