# Supplementary material for: Km-scale coupled simulation and model–observation SST trend discrepancy
Source: Proc Natl Acad Sci U S A. 2026 Feb 19;123(8):e2522161123. doi: 10.1073/pnas.2522161123 (PMC12933147; doi:10.1073/pnas.2522161123)
Supplement: Supplementary file 1 — Appendix 01 (PDF) [file pnas.2522161123.sapp.pdf]

PNAS

Supporting Information for

Km-scale coupled simulation and model-observation SST trend discrepancy.

Sarah M. Kang*, Dian A. Putrasahan, Noel G. Brizuela, Helmuth Haak, Jürgen Kröger, Jochem Marotzke, Bjorn Stevens, and Jin-Song von Storch

*Corresponding author: Sarah M. Kang

Email: sarah.kang@mpimet.mpg.de

This PDF file includes:

Figures S1 to S8
SI References

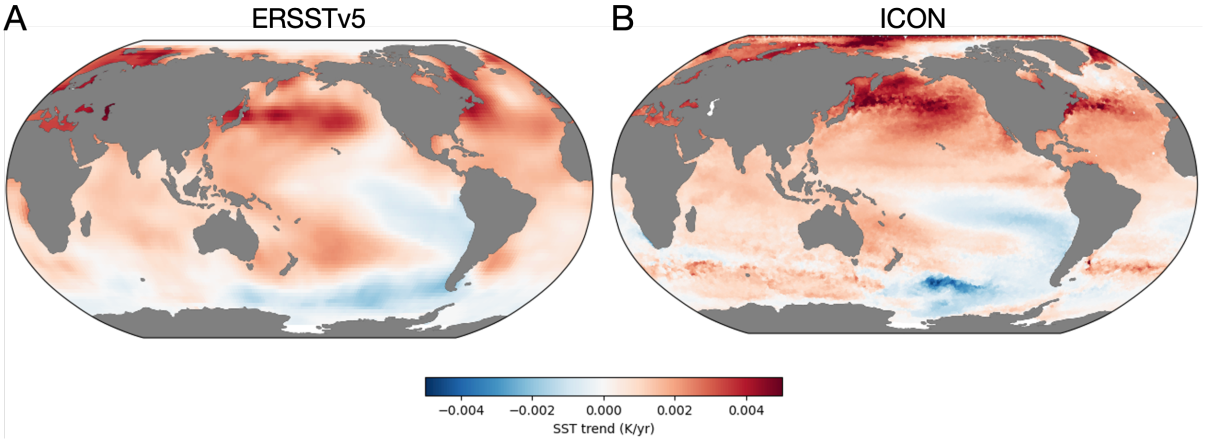

Fig. S1. Sea surface temperature trends over 1979-2024 in (*A*) ERSSTv5 and (*B*) ICON historical simulation.

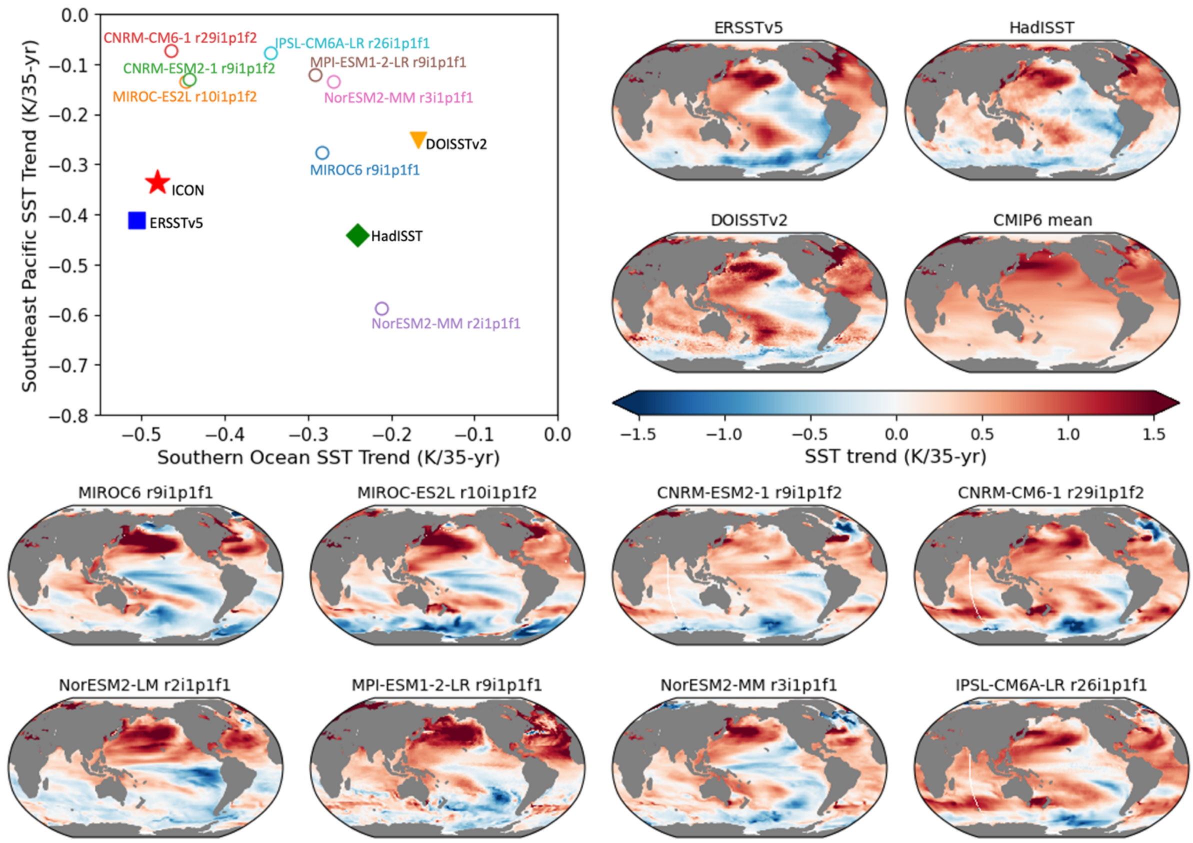

Fig. S2. The third quadrant of Fig. 2 and the spatial maps of SST trends over 1979-2014 from different observational dataset, the CMIP6 multi-model mean, and selected CMIP6 models that fall within the third quadrant.

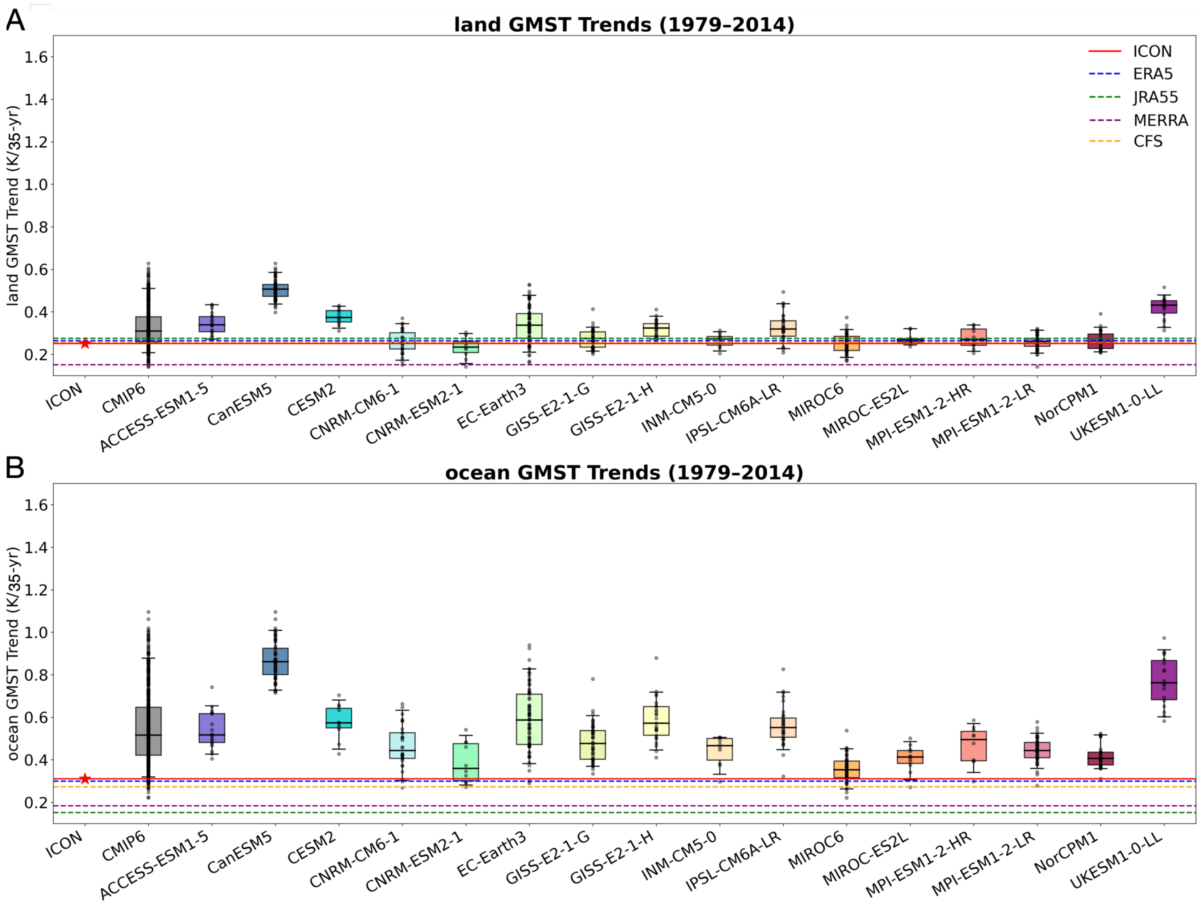

Fig. S3. Globally averaged surface temperature trends during 1979-2014 over (*A*) land and (*B*) ocean.

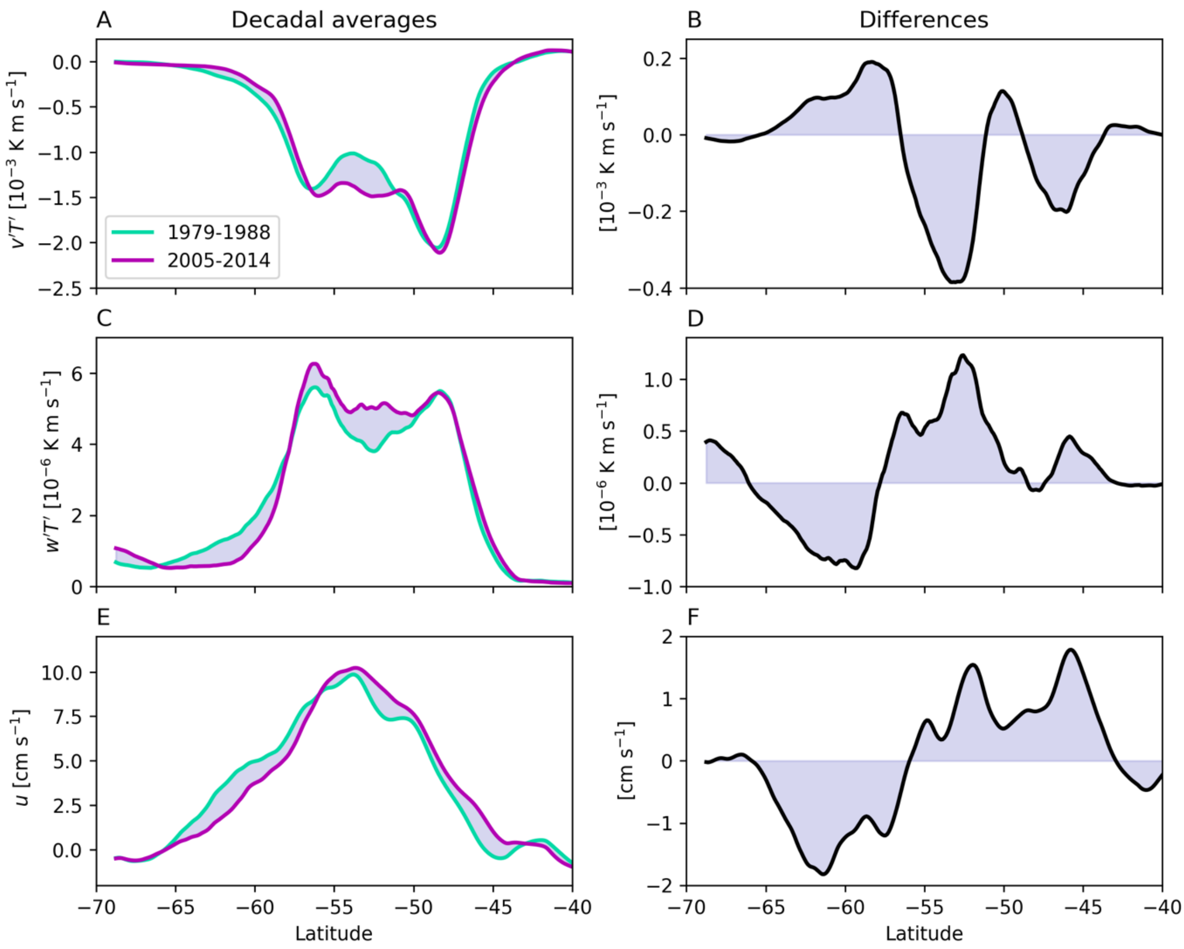

Fig. S4. Eddy temperature transports in relation to zonal ACC velocities in the Pacific sector of the Southern Ocean (180°E to 240°E). The left column shows decadal averages of (*A*) meridional and (*C*) upward temperature transport by eddies (defined as sub-monthly anomalies) and of (*E*) zonal velocities. Values are shown for the decades of 1979-1988 and 2005-2014, while corresponding differences between both decades in (*B*), (*D*), and (*F*) indicate a northward displacement in both poleward eddy heat transport and the ACC.

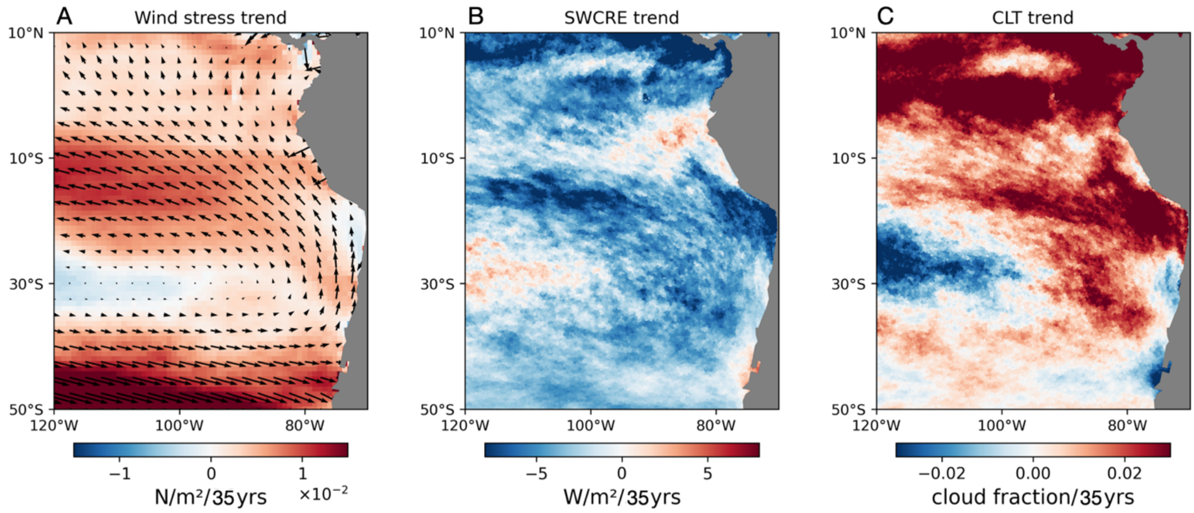

Fig. S5. Trends during 1979-2014 in (*A*) wind stress along with climatological surface wind vectors, (*B*) shortwave cloud radiative effects, and (*C*) total cloud fraction.

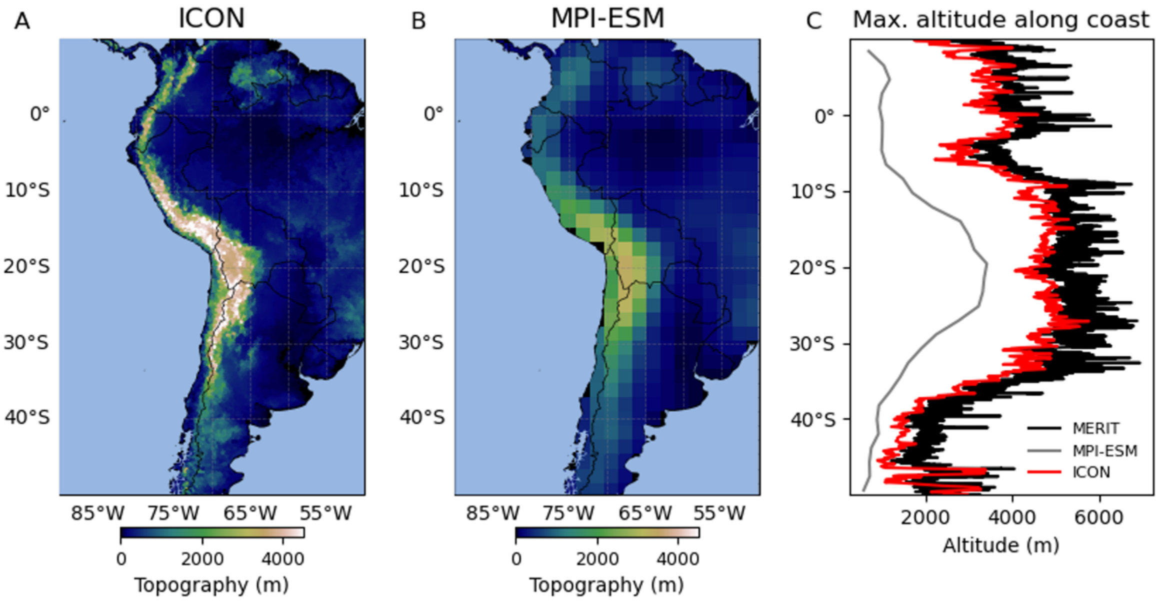

Fig. S6. Surface height across South America (in m) for (*A*) ICON and (*B*) MPI-ESM, which participated in CMIP6. (*C*) Zonal maximum surface height across South America for the observation (black), ICON (red), and MPI-ESM (gray). Observational dataset is from (1).

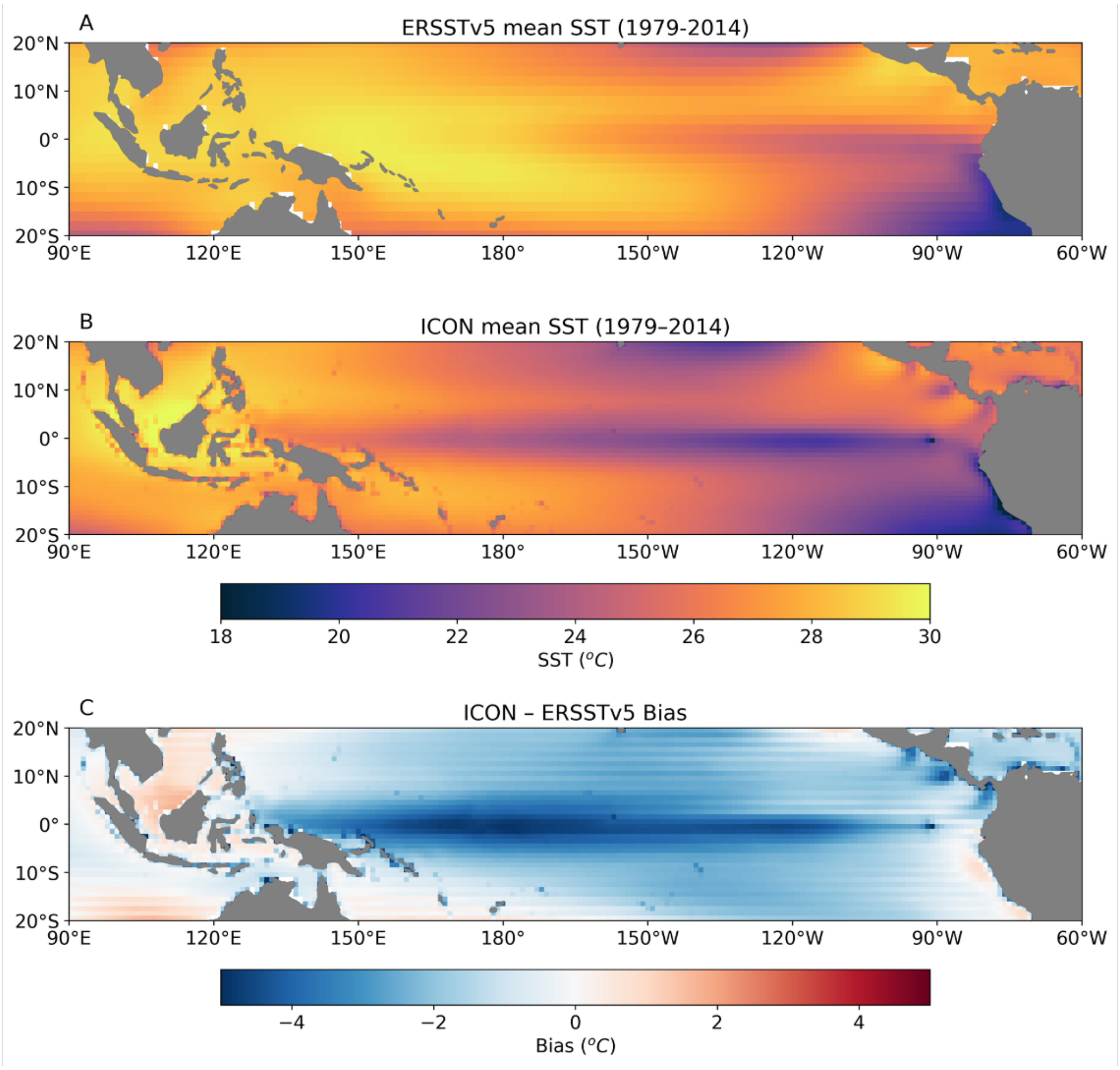

Fig. S7. Climatological mean sea surface temperature averaged over 1979-2014 from (*A*) ERSSTv5 dataset, (*B*) ICON historical simulation, and (*C*) their difference, indicating the model bias.

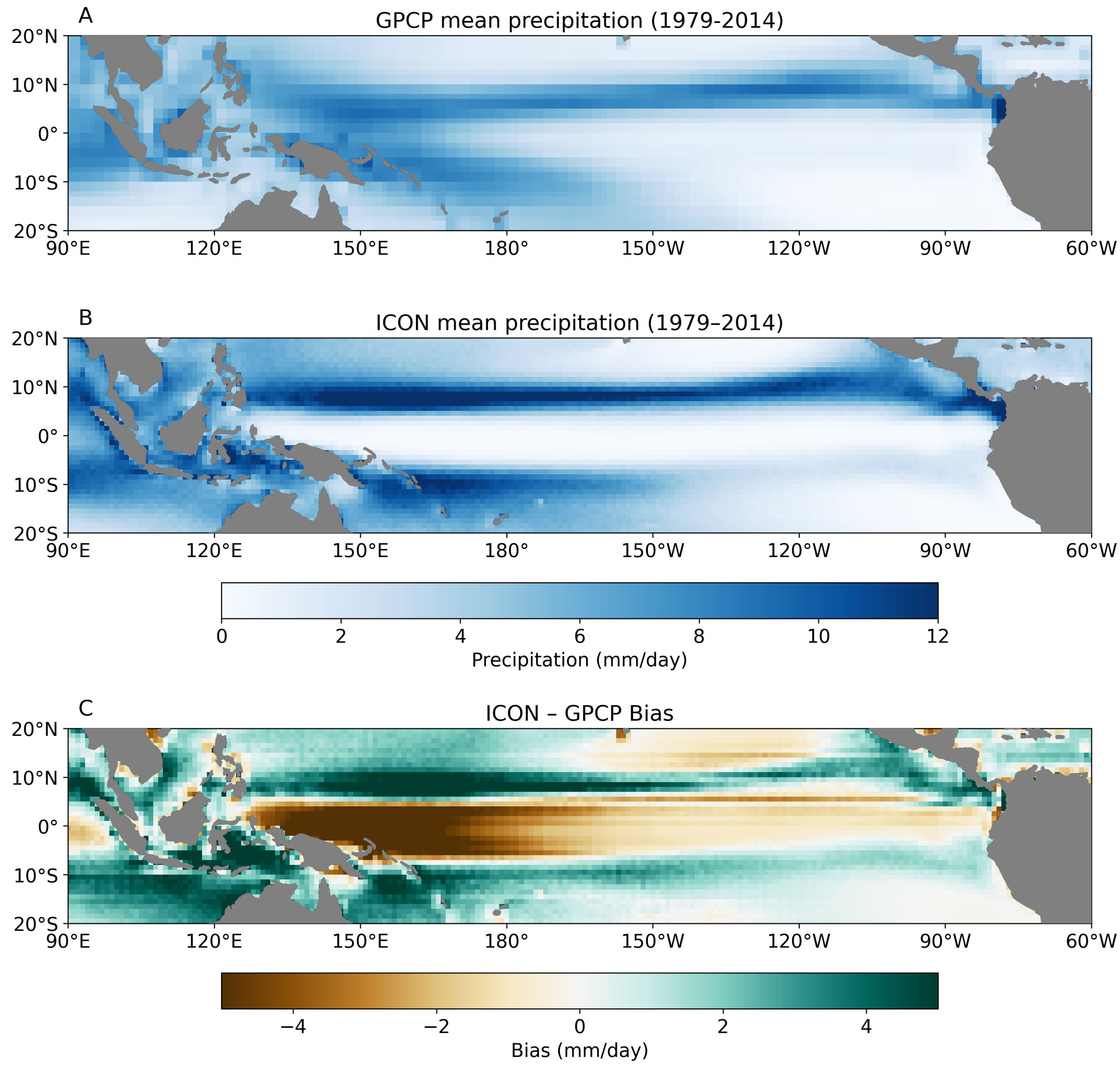

Fig. S8. Climatological mean precipitation averaged over 1979-2014 from (*A*) Global Precipitation Climatology Project (GPCP) observational dataset, (*B*) ICON historical simulation, and (*C*) their difference, indicating the model bias.

SI References

1. D. Yamazaki, *et al.*, A high-accuracy map of global terrain elevations. *Geophys. Res. Lett.* **44**, 5844–5853 (2017).
